# Promoting vascular stability through Src inhibition and Tie2 activation: A model-based analysis

**DOI:** 10.1016/j.isci.2025.112625

**Published:** 2025-05-09

**Authors:** Yu Zhang, Christopher D. Kontos, Brian H. Annex, Aleksander S. Popel

**Affiliations:** 1 Department of Biomedical Engineering, School of Medicine, Johns Hopkins University, Baltimore, MD 21215, USA; 2 Department of Medicine, Duke University Medical Center, Durham, NC 27710, USA; 3 Department of Medicine, Augusta University, Augusta, GA 30912, USA

**Keywords:** Natural sciences, Biological sciences, Bioinformatics, Pharmacoinformatics

## Abstract

Dysregulated angiogenesis signaling leads to pathological vascular growth and leakage, and is a hallmark of many diseases including cancer and ocular diseases. In peripheral arterial disease, the concomitant increase in vascular permeability presents significant challenges in therapeutic efforts to improve perfusion by stimulating vascular growth. Building a mechanistic understanding of the endothelial control of vascular growth and permeability signaling is crucial to guide our efforts to identify therapeutic strategies that permit blood vessel growth while maintaining vascular stability. We develop a mechanistic systems biology model of the endothelial signaling network formed by the vascular endothelial growth factor (VEGF) and angiopoietin (Ang)-Tie pathways, two major signaling pathways regulating vascular growth and stability. Our model, calibrated and validated against experimental data, reveals the mechanisms through which chronic Ang1 stimulation protects endothelial cells from VEGF-induced hyperpermeability, and predicts that combining Src inhibition with Tie2 activation can inhibit vascular leakage without disturbing angiogenesis signaling.

## Highlights

• Endothelial signaling model integrating VEGF and Ang-Tie pathways
• Chronic Ang1 stabilizes vessels but cannot reverse VEGF-induced leakage
• Src inhibition with Tie2 activation reduces leakage without blocking angiogenesis
• Model guides therapies to promote the growth of stable vasculature

## Graphical abstract

**Figure:**
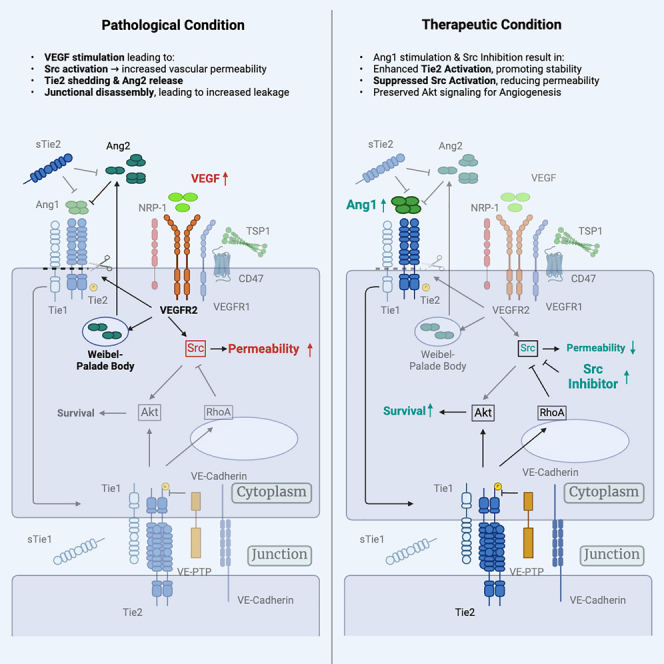

## Introduction

Angiogenesis refers to the growth of new vasculature from existing one, and it is an important physiological process in development, skeletal muscle exercise, wound healing, and reproduction.^1^ Pathological angiogenesis is characterized by the dysregulation of angiogenesis signaling that leads to the development of unstable and leaky vasculature and is a hallmark of many angiogenesis-related disease, including cancer and ocular diseases.^2^ Tumor angiogenesis is a hallmark of cancer progression and therapeutic agents to halt tumor growth by inhibiting angiogenesis have been widely used in the treatment of many types of solid tumors.^3^^,^^4^ In ocular diseases like neovascular age-related macular degeneration and diabetic retinopathy, leaky retinal vasculature causes edema and vision impairment. Therapeutic strategies to treat these diseases aim to inhibit vascular permeability and neovascularization.^5^ Another angiogenesis-related disease is peripheral arterial disease (PAD), which affects the lives of over 10 million patients in the United States and over 200 million worldwide.^6^ PAD is characterized by atherosclerotic blockades that lead to obstructed blood flow to the leg tissues, causing ischemic reperfusion injuries and tissue necrosis. Extensive research aiming to therapeutically induce angiogenesis to aid in the recovery of blood perfusion in ischemic tissues in PAD patients has had limited success.^7^ Therapeutically inducing angiogenesis with exogenous growth factors or gene therapy approaches often leads to the development of unstable, leaky vasculature that cannot provide adequate benefit to perfusion recovery,^8^ partly because many signaling pathways that promote angiogenesis also induce vascular leakage.^9^ There is still a large unmet need to discover therapeutic strategies that promote vascular growth without causing leakage to improve perfusion to ischemic tissues in patients with PAD. In what follows we will often refer to PAD applications, however, it should be noted that the analysis is general and applies to other angiogenesis-dependent diseases including cancer and ocular diseases.

Angiogenesis is a highly regulated process orchestrated and mediated by endothelial cells, perivascular cells, muscle cells and other parenchymal cells, and immune cells.^10^ Endothelial cells are major driver of angiogenesis, and they regulate vascular growth, permeability, stability, and quiescence with complex intracellular signaling networks formed by multiple signaling pathways.^11^ The vascular endothelial growth factor (VEGF) signaling pathway is one of the major endothelial signaling pathways regulating angiogenesis and vascular permeability.^12^ VEGF signaling pathway consists of ligands VEGF-A, VEGF-B, VEGF-C, and placental growth factor and receptors VEGFR1, VEGFR2, and VEGFR3.^12^^,^^13^^,^^14^ VEGF-A is the canonical activating ligand and is the focus of this study. Activation of VEGF receptor VEGFR2 by VEGF-A is associated with endothelial survival, migration, proliferation, and permeability. VEGF’s activation of VEGFR2 is regulated by many molecular regulatory mechanisms including the association with co-receptor neuropilin-1 (NRP-1),^15^ heterodimerization of VEGFR2 with VEGFR1,^16^ thrombospondin-1 (TSP-1) and its interaction with cluster of differentiation 47 (CD47).^17^ The activation of VEGFR2 promotes endothelial survival and migration through its downstream activation of phosphoinositide 3-kinase (PI3K)/Akt and calcium and Erk-dependent sphingosine-1-phosphate (S1P) activation.^18^^,^^19^^,^^20^ VEGF-induced Src activation is also associated with increased endothelial permeability and leakage.^21^

Another major endothelial signaling pathway regulating angiogenesis and vascular stability is the angiopoietin (Ang)-Tie signaling pathway. The Ang-Tie signaling pathway consists of ligands Ang1–4, receptor Tie2, and co-receptor Tie1. Ang1 and Ang2 are the most abundant ligands relevant to angiogenesis. Ang1 is an activating ligand of Tie2, whereas Ang2 is considered a context-dependent, weak agonist of Tie2 that can compete with and antagonize Ang1’s activation of Tie2.^22^^,^^23^ Ang-Tie signaling pathway is regulated by many molecular regulatory mechanisms. Activation of Tie2 requires ligand-induced multimerization and clustering by multimeric forms of Ang1, which mostly exists in clusters of tetramers or higher oligomers, or Ang2, which exists in both low and high multimeric forms.^24^ Vascular endothelial protein tyrosine phosphatase (VE-PTP) catalyzes the dephosphorylation of active Tie2 and regulates its activity.^25^ Co-receptor Tie1 does not bind to any angiopoietins but can affect Tie2 signaling by forming heterodimers with Tie2.^26^^,^^27^ Tie1’s heterodimer formation with Tie2 can both limit Tie2’s ligand access and sustain its activation by promoting Tie2’s junctional localization.^28^^,^^29^ Both Tie1 and Tie2 also undergo constitutive and ligand-induced extracellular domain shedding.^30^^,^^31^ In confluent endothelial cells, Tie2 activation promotes endothelial survival through PI3K/Akt^32^^,^^33^ signaling and enhances junctional stability by activating RhoA-GTP that can, in turn, bind to mDia and sequester Src kinase.^34^^,^^35^

VEGF and Ang-Tie signaling pathways also have intersecting downstream signaling nodes and crosstalk mechanisms that regulate each other’s activity. VEGF can induce the extracellular domain shedding of Tie2^31^ and the release of Ang2 from Weibel-Palade bodies.^36^^,^^37^ Ang1’s activation of Tie2 leads to sequestration and inhibition of Src, a downstream signaling node of VEGF associated with vascular permeability and can activate PI3K and enhance the shared downstream activation of Akt.^35^^,^^38^ To understand how vascular growth, permeability, and stability are regulated by the complex reaction network formed by VEGF and Ang-Tie pathways, their crosstalk mechanisms, and intertwined downstream signaling network, we build an integrative systems biology model with detailed molecular mechanisms to simulate the potential signaling outcomes in physiological and pathological conditions and computationally investigate the effects of potential therapeutics targeting this signaling network.

In cancer and retinal diseases, many therapeutic development efforts focus on inhibiting the activity of VEGF signaling pathway by targeting ligands and receptors with antibodies or small molecule tyrosine kinase inhibitors.^1^^,^^5^^,^^10^ Advances in our understanding of the role of Ang-Tie pathway in regulating vascular permeability have also led to the promising developments of drugs that simultaneously target VEGF and Ang2 to inhibit both vascular permeability and growth.^39^^,^^40^^,^^41^ In PAD, the need to promote the growth of blood vessels to increase perfusion while inhibiting the concomitant increases in vascular permeability makes therapeutic development uniquely challenging.^42^ A quantitative understanding of the complex interplay between angiogenesis signaling and the molecular control of vascular permeability is crucial to guide our efforts in developing combination therapies and discovering new therapeutic avenues.

The importance of VEGF and Ang-Tie signaling pathways in regulating vascular growth and permeability and their complex regulatory mechanisms have given rise to a myriad of computational models that study different aspects of these pathways in isolation.^10^ In our previous studies, we developed computational systems biology models to quantitatively investigate different aspects of these individual signaling pathways and how potential proangiogenic therapeutics targeting them can affect the signaling outcomes. For the VEGF pathway, we have investigated its downstream calcium-dependent Erk activation,^43^ the effect of TSP1 in promoting the activation of VEGFR2 and its downstream signaling,^44^ as well as the molecular mechanisms for the proangiogenic effect of αvß3 through its binding to VEGFR2.^45^ We have also developed computational models of the Ang-Tie pathway to quantitatively investigate the molecular mechanisms that regulate the activation of Tie2 and its signaling outcomes, as well as potential therapeutic targets in the pathway that could promote vascular stability in the context of PAD.^46^^,^^47^ However, computational models that study the VEGF or Ang-Tie pathway in isolation do not take into account the complex interplay and crosstalk mechanisms between the two pathways and the integrated downstream signaling network formed by them. In this study, we combine our systems biology understanding of VEGF and Ang-Tie models obtained from previous studies with new mechanistic findings of the crosstalk and the intersecting downstream signaling network formed by these two pathways to build an integrative model of the endothelial signaling network to quantitatively characterize the molecular control of vascular growth, permeability, and stability. Our model mechanistically explains the vascular protective function of Ang-Tie’s chronic stimulation and demonstrates that acute activation of Tie2 by Ang1 is not sufficient to reverse VEGF-induced hyperpermeability. The model also quantitatively predicts the effect of Src inhibition in suppressing vascular permeability and its inhibition of angiogenesis signaling, which could be rescued by combination with Tie2 activation.

## Results

### Integrative systems biology of the endothelial cell signaling network predicts the effects of VEGF and Ang-Tie signaling pathways

**Figure 1:**
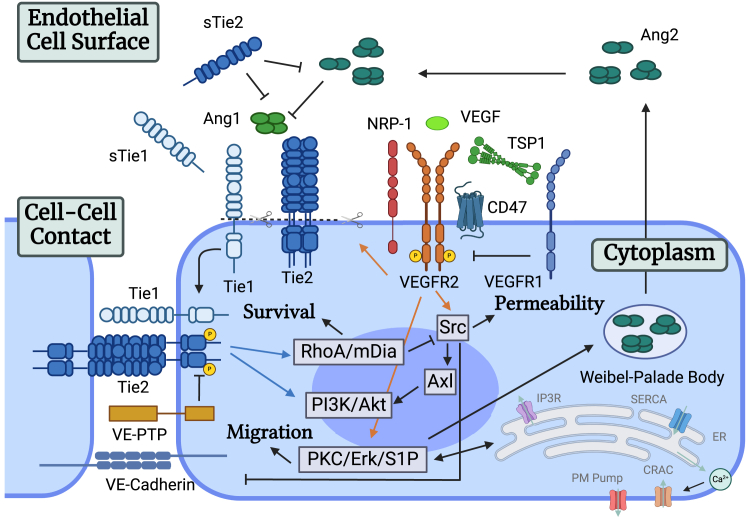
Diagram of the endothelial signaling network consisting of VEGF and Ang-Tie pathways The model mechanistically incorporates Ang1- and Ang2-induced multimerization and activation of Tie2, Tie2’s junctional location and heterodimer formation with Tie1, Tie receptors’ extracellular domain shedding, activation of VEGFR2 by VEGF, modulation of VEGFR2 by co-receptor NRP1 and VEGFR1, and the interaction of TSP1/CD47 with VEGFR2. Created with biorender.com.

We construct a computational systems biology model of the endothelial signaling network formed by VEGF and Ang-Tie signaling pathways. In the model, molecular species and reaction rules of the VEGF signaling pathways are adapted from our previously calibrated and validated mechanistic computational models of this pathway.^44^^,^^48^ In short, VEGF receptor VEGFR2 forms a homodimer that is activated by autophosphorylation upon binding to its ligand VEGF. The model also assumes that VEGFR1 acts as a decoy receptor and can form a heterodimer with VEGFR2. Co-receptor NRP1 can also affect VEGFR2 dynamics by associating with VEGFR1 and VEGF. TSP1 can bind to CD47, allowing for its association with VEGFR2. The model also incorporates the internalization, recycling, synthesis, and degradation of VEGFR2. In the Ang-Tie signaling pathway, the multimerization and activation of Tie2 receptors are associated with vascular quiescence and stability. The model adapts and extends from our previously developed models of the Ang-Tie pathway and incorporates detailed molecular mechanisms of the activation and regulation of Tie2.^46^^,^^47^ The Ang1 molecules, which are strong agonists of Tie2, are assumed to be in tetrameric form, whereas Ang2 molecules are assumed to exist in dimeric, trimeric, and tetrameric forms. Tetrameric Ang1 and Ang2 molecules can induce the clustering, multimerization, and subsequent activation and junctional localization of Tie2. Tie2 activity is modulated by various molecular regulatory mechanisms. Co-receptor Tie1 does not bind to any angiopoietins but can form heterodimers with Tie2 and inhibit Tie2’s ligand access at the cell surface. At the cellular junction, Tie1 can induce the co-localization of multimeric Tie2 and sustain its activation. VE-PTP regulates the activation of Tie2 by catalyzing the dephosphorylation of active Tie2 at the junctions. In addition, both Tie1 and Tie2 undergo extracellular domain shedding at the cell surface, and the cleaved product soluble Tie2 can act as a ligand trap to further inhibit Tie2 activation. An overall diagram of the model is shown in Figure 1.

**Figure 2:**
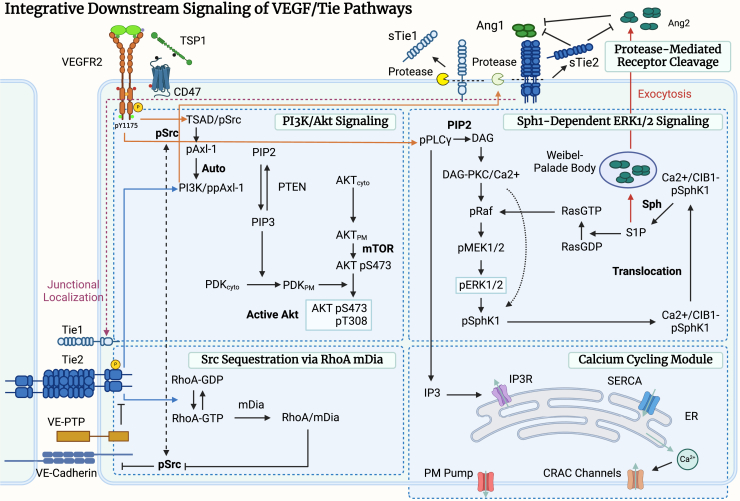
Integrative downstream signaling network of VEGF and Ang-Tie signaling pathways The downstream signaling network consists of PI3K/Akt signaling, Sph1-dependent Erk signaling, Src sequestration via RhoA/mDia, and a calcium cycling module. Created with biorender.com.

The model also incorporates detailed molecular mechanisms of the crosstalk between the VEGF and Ang-Tie signaling pathways, as well as their integrative downstream signaling network. Activation of VEGFR2 induces Src-dependent PI3K activation, which in turn leads to Akt activation and subsequent calcium-dependent eNOS activation. VEGFR2 also activates PLCγ and induces its subsequent S1P- and calcium-dependent activation of Erk through Raf/MEK. Activated PLCγ also modulates intracellular calcium concentration through IP3-induced calcium release. In addition, VEGFR2 activation induces extracellular domain shedding of Tie2 and the release of Ang2 from Weibel-Palade bodies through S1P. In the Tie2 signaling pathway, activated Tie2, once localized at the endothelial junctions, activates Akt via PI3k independent of Src. Tie2 activation also promotes the activation of RhoA-GTP, which subsequently binds to mDia, forming a complex that can bind and sequester Src, preventing its phosphorylation by VEGFR2. A detailed diagram of the integrative signaling network and the crosstalk mechanisms formed by VEGF and Ang-Tie pathways is shown in Figure 2.

**Figure 3:**
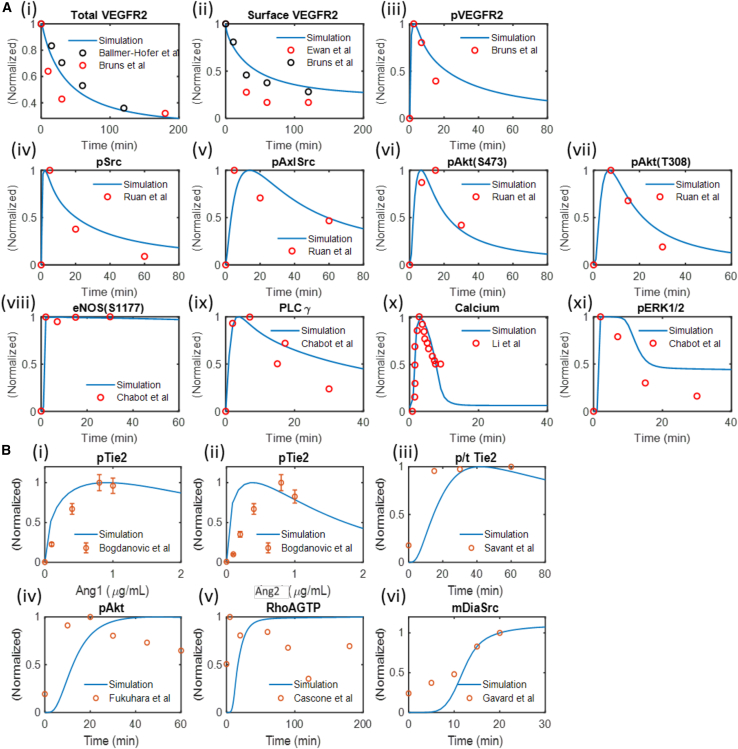
Model calibration to experimental data on VEGF and Ang-Tie pathways (A) Model calibration to experimental data on VEGF-induced VEGFR2 activation, receptor dynamics, and downstream signaling through Akt, Erk, and calcium. (B) Model calibration to experimental data on Ang1- and Ang2-induced Tie2 activation and downstream signaling.

The model is carefully calibrated to experimental data from independent sources measuring the activity of VEGF and Ang-Tie signaling pathways. Figures 3A and 3B show model simulations compared to experimental data. Figure 3A (i–iii) shows the model calibration to experimentally measured total VEGFR2,^49^^,^^50^ surface VEGFR2,^49^^,^^51^ and activated VEGFR2 (pVEGFR2) dynamics^49^ following VEGF stimulation. Figure 3A (iv–viii) shows the model calibration to experimentally measured activation of Src, Axl, and Akt following VEGF stimulation. Figure 3A (ix–xii) shows the calibration to eNOS activation, PLCγ activation, intracellular calcium concentration, and the activation of Erk1/2 by VEGF.^52^^,^^53^ Figure 3B (i and ii) shows the dose-dependent activation of Tie2 by varying concentrations of Ang1 and Ang2 calibrated to experimental results.^54^ Figure 3B (iii–vi) shows the model calibration of the activation of Tie2 and its downstream activation of Akt, RhoA-GTP, and mDia-Src complex formation dynamics following Ang1 stimulation to experimental data.^29^^,^^32^^,^^34^^,^^35^

### VEGF disrupts Ang-Tie signaling and endothelial junctional integrity by promoting Src activation, Tie2 shedding, and Ang2 release

**Figure 4:**
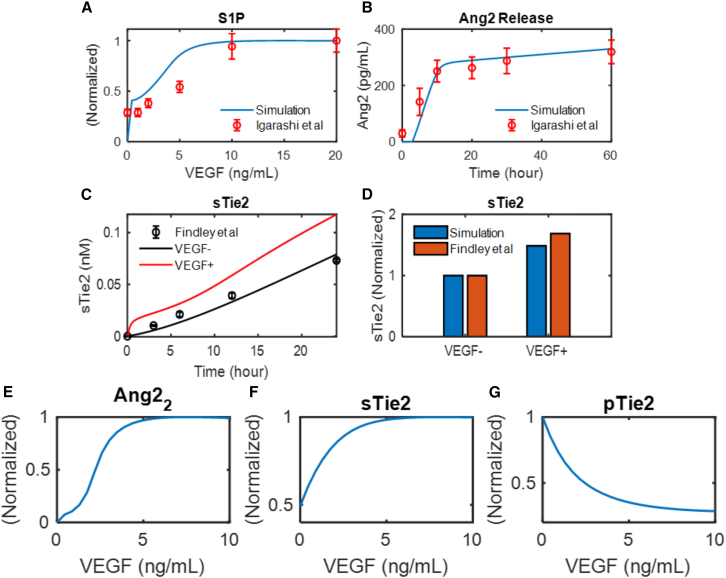
Model calibration to experimental data on VEGF’s regulation of Ang-Tie signaling (A–D) Model calibration to experimental data on VEGF-induced S1P activation, Ang2 release, constitutive Tie2 shedding, and VEGF-induced Tie2 shedding, error bars indicate the standard error of the mean. (E–G) Model prediction of the dose-dependent effect of VEGF on Ang2 release, Tie2 shedding, and Tie2 activation.

VEGF has been observed to accelerate the extracellular domain shedding of Tie2.^31^ VEGF-induced activation of S1P also activates Weibel-Palade bodies and promotes the release of Ang2, which further suppresses the activation of Tie2.^36^^,^^37^ To quantitatively predict the effect of VEGF on Tie2 signaling, the model is calibrated to experimental data from independent sources, as shown in Figure 4. Figure 4A shows the model calibration to dose-dependent activation of S1P by VEGF ranging from 0 to 20 ng/mL, as compared to experimental results in a study by Igarashi et al.^37^ Figure 4B shows the model calibration to the release of Ang2 over time following S1P activation, compared to experimental data reported in a study by Jang et al.^36^ In Figure 4C, the model is calibrated to experimental data measuring the constitutive shedding of Tie2 without VEGF stimulation, and Figure 4D shows the model calibration to the effect of VEGF in promoting the extracellular domain shedding of Tie2, as reported in a study by Findley et al.^31^

The calibrated model is then used to quantitatively predict the dose-dependent effect of VEGF on the release of Ang2, Tie2 shedding, and the activation of Tie2. Figures 4E and 4F shows that the model predicts that VEGF-induced Ang2 release and Tie2 shedding saturates at around 7 ng/mL of Ang2 concentration. VEGF enhances the shedding of Tie2 to up to 2-fold of its constitutive shedding (Figure 4F). High VEGF presence at more than 7 ng/mL is predicted to inhibit Ang1-induced Tie2 activation by up to 90% (Figure 4G). Tie2 shedding and Ang2 overexpression have also been observed *in vivo* in serum of patient with angiogenesis-related diseases.^55^^,^^56^ The model provides a quantitative and mechanistic explanation for VEGF’s disruptive effect on Tie2 activation, inducing vascular permeability and destabilization, and is used to simulate the pathological signaling outcomes of endothelial cells with heightened VEGF, Tie2 shedding, and Ang2, as well as the effects of perturbations to these cells.

### Global sensitivity analysis identifies key mechanisms affecting Src and Akt activation

**Figure 5:**
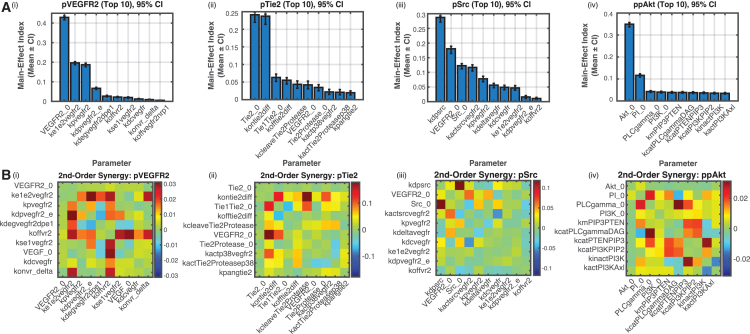
Global sensitivity analysis (A) Bar plots showing the top 10 parameters ranked by their main-effect indices with 95% confidence intervals (CI) for: (i) phosphorylated VEGFR2 (pVEGFR2), (ii) phosphorylated Tie2 (pTie2), (iii) phosphorylated Src (pSrc), and (iv) phosphorylated Akt (ppAkt). These indices represent the individual contributions of parameters to the variance in the respective pathway outputs, error bars indicate the standard error of the mean. (B) Heatmaps depicting second-order synergy indices for parameter pairs, which highlight interactions contributing to the variance in pathway outputs: (i) pVEGFR2, (ii) pTie2, (iii) pSrc, and (iv) ppAkt. Warmer colors indicate stronger synergistic effects, while cooler colors indicate less significant interactions. Key parameters with substantial effects include receptor phosphorylation rates, ligand-receptor interactions, and downstream signaling enzyme kinetics.

The key signaling outcomes of the reaction network formed by VEGF and Ang-Tie signaling pathways, in addition to the activation of receptors VEGFR2 and Tie2, include activation of Akt, which is linked to endothelial survival, and activation of Src, which is known to induce hyperpermeability. To identify the key parameters that influence these signaling outcomes, we perform a global sensitivity analysis using Sobol indices, focusing on both first-order and second-order effects. The analysis involves sampling the parameter space with Latin hypercube sampling (LHS) and calculating Sobol main-effect (first-order) indices and second-order synergy indices for all model parameters, as detailed in the STAR Methods section. The results in Figure 5A highlight the top 10 parameters with the greatest influence on the signaling outcomes pVEGFR2, pTie2, pSrc, and ppAkt.

The Sobol first-order analysis also suggests that ppAkt activation is most sensitive to parameters associated with PI3K-Akt signaling, including PLCγ (PLCgamma_0, kcatPLCgammaDAG), PI3K activation (kinactPI3K, kcatPI3KAkt), and the VEGFR2 axis (ksynvegfr2, kdegvegfr2). Similarly, pSrc activation is strongly influenced by Src activity (Src_0, kactivesrc), VEGFR2 parameters (ksynvegfr2, koffvegfr2), and Tie2 degradation (kdegptie2). For pTie2, key parameters include Tie2 availability (Tie2_0) and ligand-receptor interactions (konang1tie2, koffang2tie2). Finally, pVEGFR2 is primarily affected by VEGFR2 synthesis and degradation rates (ksynvegfr2, kdegvegfr2dpe2_nrp) and ligand-receptor binding kinetics. The Sobol second-order synergy analysis (Figure 5B [i–iv]) highlights interactions between parameters that significantly influence signaling outcomes. For instance, synergistic interactions between VEGF-mediated VEGFR2 activation and Ang2-induced Tie2 activation are critical for ppAkt activation. Similarly, interactions involving Src and VEGFR2 pathways play a dominant role in pSrc activation. For pTie2 and pVEGFR2, strong synergistic effects are observed in parameters governing ligand-receptor dynamics and receptor trafficking, emphasizing their importance in fine-tuning signaling responses.

These findings suggest that targeting key nodes, such as VEGFR2 and Tie2, can modulate multiple signaling outcomes simultaneously. Our model supports the current therapeutic focus on VEGF and Ang2 inhibitors, which suppress both vascular permeability and Akt-mediated angiogenic signaling. While this dual effect is desirable for cancer and ocular disease, it presents challenges in PAD, where the goal is to suppress hyperpermeability while maintaining angiogenic signaling. The results suggest alternative strategies, such as inhibiting Src activation or promoting the activation of Tie2, to achieve selective suppression of vascular permeability without disrupting Akt-mediated angiogenesis.

### Chronic Ang1 stimulation protects endothelial cells from VEGF-induced hyperpermeability; Ang1 stimulation alone cannot reverse acute VEGF-induced hyperpermeability

**Figure 6:**
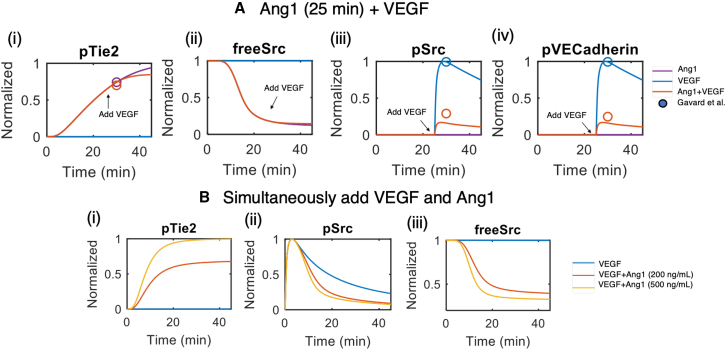
Model calibration to experimental data on VEGF’s regulation of Ang-Tie signaling (A(i)–(iv)) Model calibration to experimental data on VEGF-induced S1P activation, Ang2 release, constitutive Tie2 shedding, and VEGF-induced Tie2 shedding. (B(i)–(iii)) Model prediction of the dose-dependent effect of VEGF on Ang2 release, Tie2 shedding, and Tie2 activation.

Ang1-stimulated activation of Tie2 is associated with vascular stability and quiescence. Experimental studies in Cascone et al. and Gavard et al. show that Ang1-induced downstream activation of RhoA promotes the formation of RhoA-GTP/mDia complex, which in turn binds to and sequesters Src, a kinase associated with vascular permeability.^34^^,^^35^ We use our calibrated model to simulate the effect of Ang1 stimulation on VEGF-induced permeability and compare the results to experimental data not used in model calibration in Gavard et al. to validate the model.^35^ In these simulations, we compare the signaling outcomes of stimulating the endothelial cells with 50 ng/mL of VEGF to the signaling outcomes of pre-stimulating the endothelial cells with 50 ng/mL of Ang1 for 25 min and subsequently adding VEGF. We also simulated the signaling outcomes of simulating with only Ang1 without VEGF addition. Consistent with experimental findings, our simulations show that Ang1 through activating Tie2 can sequester Src, reducing the amount of free Src available for VEGF to activate. Stabilizing the endothelial cells with 25 min of Ang1-stimulation leads to a marked 80% decrease in maximum Src phosphorylation and VE-Cadherin phosphorylation after VEGF is added. Both Src and VE-Cadherin phosphorylation are associated with vascular hyperpermeability and leakage.^57^^,^^58^ Simulation predicts that adding VEGF 25 min post Ang1 stimulation has little effect on Tie2 activation (Figure 6Ai). Consistent with experimental data,^35^ our simulations also demonstrate that chronic Ang1 stimulation stabilizes endothelial junctions by sequestering Src and protects the endothelial cells from acute VEGF-induced hyperpermeability. Our simulation shows that 25 min of Ang1 stimulation at 50 ng/mL results in high Tie2 activation and up to 90% of Src sequestration, and Ang1 stabilization reduces both VEGF-induced maximum Src and VE-Cadherin activation by approximately 85% (Figure 6A [ii–iv]).

Our model demonstrates the effect of Ang1 in stabilizing endothelial cells and protecting them from VEGF-induced hyperpermeability, consistent with experimental data. We then use the validated model to test if adding Ang1 simultaneously with VEGF can reverse the vascular leakage. Our simulations predict that Ang1 can still induce a dose-dependent Tie2 activation. However, Ang1 alone fails to have an inhibitory effect on maximum acute Src activation induced by VEGF, even at high concentrations of 200 and 500 ng/mL. The model shows that mechanistically, Ang1-induced Src sequestration reaches its maximum effect after 20 min post-stimulation. VEGF-induced Src activation, however, happens rapidly and reaches maximum activation of Src at approximately 5 min post-stimulation, before Ang1-induced Src sequestration comes into effect. These results suggest that chronic Ang1 stimulation can stabilize endothelial cells and protect them against VEGF-induced hyperpermeability. However, if endothelial cells are already in a hyperpermeable state, acute Ang1 stimulation is not sufficient to reverse VEGF-induced permeability.

### Src inhibition combined with Ang1 stimulation promotes junctional stability and angiogenesis

**Figure 7:**
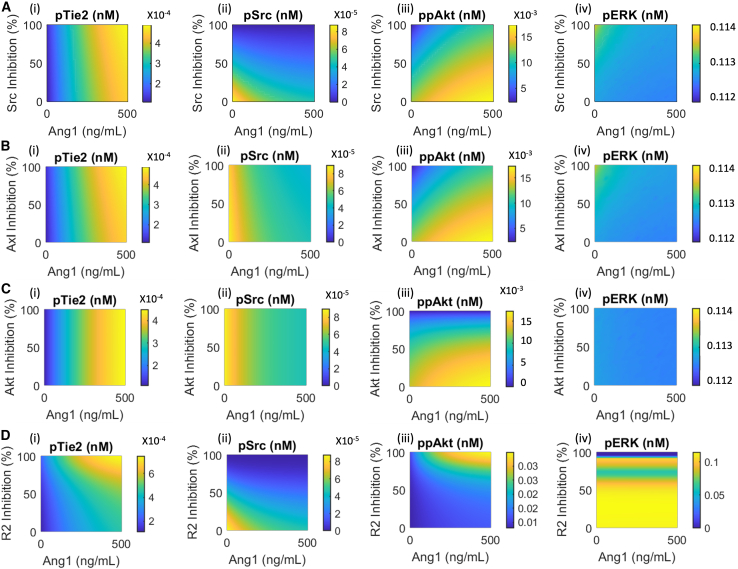
Model predictions of the effects of combining Ang1 stimulation with inhibitors targeting VEGFR2/Src/Akt axis Predicted pTie2, pSrc, ppAkt, and pErk at 15 min post stimulation following simultaneous stimulation with Ang1 and (A) Src inhibitor, (B) Axl inhibitor, (C) Akt inhibitor, and (D) VEGFR2 inhibitor.

In cancer, ocular disease and PAD patients, elevated levels of VEGF and Ang2 are associated with worse outcomes,^55^^,^^59^^,^^60^^,^^61^ likely causing endothelial cells to exhibit chronic pro-permeability signaling. Simulations using our validated model suggest that in this scenario, Ang1 alone is not sufficient to reverse VEGF-induced Src activation and hyperpermeability. Inhibition of Src activation can be alternatively achieved by using small molecule inhibitors that target Src or its upstream signaling nodes, such as VEGFR2. Indeed, inhibitors of Src and VEGFR2 have been observed to reduce vascular permeability.^62^^,^^63^ These inhibitors, however, disrupt angiogenesis signaling and do not benefit patients of PAD. Because of Ang1’s ability to enhance VEGF-induced Akt activation and endothelial survival, we computationally investigate the signaling outcomes of combining Ang1 stimulation with inhibitors of Src, Akt, Axl, and VEGFR2. Our simulation shows that Src inhibition effectively inhibits Src activation but results in the downstream inhibition of Akt that can be rescued by Ang1 stimulation (Figure 7A). The combination of moderate Src inhibition with 500 ng/mL of Ang1 can effectively inhibit Src activation without disrupting Tie2, Akt, and Erk signaling (Figure 7A).

Inhibition of Axl or Akt, however, even in combination with Ang1, cannot inhibit Src activation as effectively as direct Src inhibition (Figures 7B and 7C). Our model further demonstrates that if Akt is inhibited by more than 60%, Ang1 addition cannot effectively rescue Akt activation (Figure 7C). Finally, our model predicts that VEGFR2 inhibition synergistically activates Tie2 with Ang2 stimulation, and its inhibitory effect on Akt can still be rescued by Ang1 addition when the inhibition level of VEGFR2 is high, its inhibition of Erk, however, cannot be rescued by Ang1 addition and will lead to suppressed endothelial migration signaling through Erk (Figure 7D).

## Discussion

The model we present in this study integrates and extends our previously published models of VEGF signaling pathway and its downstream signaling through Akt and Erk,^43^^,^^44^^,^^48^ and our models of Ang-Tie signaling pathway.^46^^,^^47^ The integrated model focuses on the crosstalk mechanisms through which the two signaling pathways regulate and affect each other’s signaling and can be used to simulate the effect of simultaneously perturbing both VEGF and Ang-Tie pathways. The main signaling outcomes in this signaling networks are Akt,^64^ which is associated with endothelial cell survival, Erk, which is associated with endothelial cell migration,^65^ and Src and VE-cadherin, which are associated with vascular permeability.^21^^,^^62^^,^^66^

Ang1’s activation of Tie2 has been known to promote vascular stability and quiescence. Therapeutic drugs targeting this pathway typically aim to promote the activation of Tie2.^67^^,^^68^ Cartilage oligomeric matrix protein (COMP)-Ang1 is an engineered potent variant of Ang1 that promotes the multimerization and activation of Tie2.^69^ Ang2-binding Tie2-activating antibody (ABTAA) promotes Tie2 activation by promoting the agonistic activity of Ang2.^70^ AKB9778, a small molecule inhibitor of VE-PTP, has also been demonstrated to promote the activation of Tie2.^25^ Ang2 antibodies have also been developed to inhibit Ang2’s antagonistic effect on Tie2 activation by Ang1 and have been demonstrated to be effective when used in combination with VEGF inhibition to inhibit angiogenesis and vascular leakage. In PAD, however, the signaling objective is to promote the growth of stable vasculature. This means that effective therapeutic strategies in PAD should simultaneously inhibit vascular leakage and promote other angiogenesis signaling. In our simulations, we demonstrate that although Ang1 has a marked effect of stabilizing endothelial cells and protecting them from VEGF-induced permeability, this effect requires chronic stimulation by Ang1 without the presence of VEGF. If the endothelial cells are already in a pro-angiogenic and permeable state, Ang1 stimulation alone or other therapeutic strategies that aim to promote the activation of Tie2 are likely insufficient to reverse vascular leakage. In PAD patients, serum concentrations of VEGF and Ang2 have been observed to be elevated.^55^^,^^71^ This could cause the endothelial cells to exhibit pro-permeability signaling that is unable to be reversed by Tie activation, resulting in pro-angiogenic therapies to induce the growth of leaky, and not stable, vasculature. Based on our model predictions, we hypothesize that it is beneficial to condition endothelial cells to be in a pro-stability and quiescent state before inducing angiogenesis to promote the growth of stable vasculature.

Using the model, we further investigate the signaling outcomes of using Ang1 stimulation in combination with other therapeutics targeting the endothelial signaling network. Most therapeutics targeting the endothelial cells in cancer and ocular diseases, including many tyrosine kinase inhibitors targeting different nodes of this signaling network, aim to inhibit angiogenesis signaling, which is contrary to the therapeutic objective in PAD.^48^^,^^72^ Examples in this class of drugs include sunitinib, a VEGFR2 inhibitor,^73^ dasatinib, an inhibitor of breakpoint cluster region and Src,^74^ and LY294002, an Akt inhibitor.^75^ Inhibitors of VEGFR2, Src, or Akt, for example, diminish Akt activation, which is associated with endothelial cell survival. VEGFR2 inhibitors further inhibit downstream signaling through Erk, which is essential for migration during angiogenesis. In addition to targeting endothelial cells, Src inhibitors also effectively target cancer cells to suppress cell cycle progression, survival, and proliferation and are being used in treatment of leukemia with clinical trials for many types of solid tumors ongoing.^76^ Ang1’s ability to sequester Src while activating Akt independent of it makes it desirable to be used in combination with Src inhibitor. While Src inhibitor causes downstream inhibition of Akt, Ang1 can partially rescue Akt activation through PI3K and simultaneously promote vascular stability.

Our model mechanistically demonstrates Ang1’s and Src inhibitors’ vascular protective and anti-permeability effects, which is an essential therapeutic goal in ocular diseases and cancer. Tie2 activation also has been shown to promote the normalization of tumor vasculature, leading to improved drug delivery and tumor microenvironment.^70^ In addition to inhibiting vascular leakage, promoting the growth of stable vasculature is also important to improve therapeutic benefits in PAD patients. Our model suggests that simultaneously promoting Tie2 activation and inhibiting Src can achieve the desired signaling outcome. We note that although the *in vitro* signaling responses as predicted by the model and validated by experiments reach steady states 30 min to an hour post-stimulation, the *in vivo* development of stable vasculature for therapeutic angiogenesis to provide prefusion benefits in PAD can take weeks to months of time.^77^ Further experimentation is required to assess the long-term, *in vivo* efficacy of Tie2 activation and Src inhibition. In addition to the signaling network formed by VEGF and Ang-Tie pathways, other endothelial signaling pathways including interleukin- (IL)-6, Notch, fibroblast growth factor, and hepatocyte growth factor have been explored as targets for proangiogenic therapies.^78^^,^^79^^,^^80^^,^^81^ In addition to endothelial cells, other cells in the angiogenesis microenvironment can affect the efficacies of proangiogenic therapies in growing stable vasculature. Macrophages and their phenotypic switch have been extensively studied in the context of PAD.^82^^,^^83^ Developing successful therapeutic strategies in PAD to promote the growth of stable vasculature to facilitate perfusion recovery can benefit from our progressive integration of the mechanistic understanding we obtain from these systems biology models.

Our computational model provides mechanistic insights to the regulation of vascular growth and stability through the endothelial VEGF and Ang-Tie pathways, and future work should focus on *in vitro* and *in vivo* experimental validations of the predictions. Recent advancements in *in vitro* experimental techniques including microfluidic platforms allow precise control of shear stress, ligand gradients, and endothelial barrier function, enabling quantitative validation of key signaling mechanisms identified in our model.^84^ Using microfluidic-based systems and organ-on-a-chip platforms,^84^^,^^85^ the time-dependent effect of Ang1 preconditioning on VEGF-stimulation can be validated and the effect of Src inhibition could be tested *in vitro*.

In conclusion, we have developed an integrative mechanistic systems biology model of the endothelial cell signaling network formed by the VEGF and Ang-Tie signaling pathways that regulate vascular growth and stability. Our model is carefully calibrated and validated against experimental data and provides a mechanistic hypothesis of the time-dependent effect of Ang1 on vascular permeability. Our model shows that chronic Ang1 stimulation can effectively protect endothelial cells from VEGF-induced hyperpermeability, but acute Ang1 stimulation alone is not sufficient to revert VEGF-induced leakage. The model also predicts that combining Src inhibition with Ang1 stimulation can inhibit vascular permeability without diminishing other aspects of angiogenesis signaling.

### Limitations of the study

The computational model in this study is developed based on *in vitro* experimental data and conditions. Model assumptions and simplifications may not fully capture *in vivo* endothelial signaling complexity and its integration with other angiogenesis-related pathways. Future research should focus on expanding pathway integration, validation predictions with experimental data, and conducting *in vivo* studies.

## Resource availability

### Lead contact

Any data requests should be directed to the lead contact, Yu Zhang (zhangyu@jhmi.edu).

### Materials availability

This study did not generate new biological materials.

### Data and code availability

The computational model developed in this study is available in the supplemental file (systems biology markup language [SBML] format). Additional datasets, model parameters, and MATLAB scripts for analysis are provided as supplemental materials. Any further data requests should be directed to the corresponding author.

## Acknowledgments

This work is supported by 10.13039/100000002National Institutes of Health grants 2R01EY028996-05A1 (A.S.P.), R01HL101200 (A.S.P. and B.H.A.), and R01HL141325 (B.H.A.). The authors are grateful to Dr. Feilim Mac Gabhann for critical comments and constructive suggestions. Part of this work was carried out at the Advanced Research Computing at Hopkins (ARCH) core facility (rockfish.jhu.edu), which is supported by the 10.13039/100000001National Science Foundation (NSF) grant number OAC1920103.

## Author contributions

Y.Z., B.H.A., and A.S.P. conceptualized and designed the study. Y.Z. constructed the signaling model and performed all model analyses. A.S.P. and B.H.A. provided critical input and jointly supervised the study. Y.Z. drafted the manuscript. All authors reviewed and edited the manuscript.

## Declaration of interests

Y.Z.’s current affiliation is Wyss Institute at Harvard University, Boston, MA; Broad Institute of MIT and Harvard, Cambridge, MA; Massachusetts Institute of Technology, Cambridge, MA. A.S.P. is a co-founder of AsclepiX Therapeutics and Terebra Therapeutics. He has served as a consultant to Janssen/J&J and Incyte. He receives research funding from Merck. The terms of these arrangements are being managed by Johns Hopkins University in accordance with its conflict of interest policies.

## Declaration of generative AI and AI-assisted technologies in the writing process

During the preparation of this work the authors used ChatGPT in order to improve the language and readability of the text. After using this tool/service, the authors reviewed and edited the content as needed and take full responsibility for the content of the publication.

## STAR★Methods

### Key resources table

**Table:** 

REAGENT or RESOURCE	SOURCE	IDENTIFIER
**Deposited data**		

SBML model	Developed in this study.	Data S1.

**Software and algorithms**		

BioNetGen	https://bionetgen.org/	BioNetGen-2.2.5-stable
MATLAB	Mathworks, Natick, MA	R2022a
SUNDIALS	https://computing.llnl.gov/projects/sundials	SUNDIALS v6.2.0

**Other**		

High-performance Computing Cluster	ARCH, Johns Hopkins University	https://www.arch.jhu.edu/

### Method details

#### Rule-based modeling of the endothelial signaling network regulating vascular growth and stability

The computational model presented in this study is developed using the rule-based language BioNetGen.^86^ The model inherits all species and reaction rules from our previously developed models of VEGF signaling pathway^43^^,^^44^^,^^48^ and Ang-Tie signaling pathway,^46^^,^^47^ and in addition, incorporates the crosstalk mechanisms and integrated downstream signaling. The detailed reactions are as follows:

The VEGF signaling module includes the VEGF ligand (VEGF-A), receptors VEGFR1 and VEGFR2, and co-receptors NRP1 and CD47. Upon ligand binding, VEGFR2 forms homodimers, which become activated via autophosphorylation. The model also incorporates VEGFR1 as a decoy receptor that can heterodimerize with VEGFR2, modulating VEGFR2’s activation. Co-receptor NRP1 interacts with VEGF and VEGFR1 to influence VEGFR2 activation dynamics. Additionally, CD47, upon binding to its ligand TSP1, can associate with VEGFR2 and regulates its activity.

The model also accounts for cellular trafficking processes including VEGFR2 internalization, recycling back to the membrane, synthesis, and degradation. Ligand binding and receptor dimerization are modeled as reversible binding events with associated phosphorylation rules. Trafficking events are implemented as first-order reactions dependent on receptor state and cellular compartment.

The Ang-Tie module includes Ang1 and Ang2 ligands, their receptor Tie2, co-receptor Tie1, and the phosphatase VE-PTP. Ang1 is modeled as a tetrameric agonist, while Ang2 exists in dimeric, trimeric, and tetrameric forms, allowing nuanced control over Tie2 activation. Tetrameric Ang1 and Ang2 can cluster Tie2 receptors, driving multimerization and phosphorylation of Tie2. These multimeric complexes also translocate to cell-cell junctions, where Tie2 activity is stabilized.

Tie1, although not a direct ligand-binding receptor, modulates Tie2 signaling by heterodimerizing with Tie2 and shielding ligand binding. At the junctions, Tie1 also facilitates co-localization and stabilization of Tie2 activation. VE-PTP dephosphorylates active Tie2 at junctional sites and limits sustained activation.

The model also incorporates extracellular domain shedding of both Tie1 and Tie2. The cleaved soluble Tie2 can act as a ligand trap, sequestering Ang1 and Ang2 in the extracellular space and further inhibiting Tie2 signaling.

In addition to these reaction rules from our previous models, the integrated model incorporates VEGF-induced extracellular domain shedding of Tie2, S1P-induced activation of Weibel-Palade bodies, and subsequent release of Ang2 to the extracellular space, active Tie2-induced Akt activation through PI3K, and the sequestration of Src by RhoA-GTP/mDia complex downstream of Tie2 activation. The model is defined and implemented in BioNetGen, and subsequent analysis is performed in MATLAB (MathWorks, Natick, MA) using the MATLAB executable file exported from BioNetGen. The numerical solutions of the ODE model are calculated using the SUNDIALS suite of differential equation solvers.^87^

The complete lists of model parameters and initial conditions, along with their best-fit values, are included in the supplemental information (Tables S1 and S2). The model is available as a supplemental file in the systems biology markup language (SBML) (Data S1).

#### Model calibration and validation of experimental data

The initial estimation of the parameters and initial concentrations of the species are inherited from our published and calibrated models of the VEGF and Ang-Tie signaling pathways. We have included all model parameters and initial conditions, along with their best-fit values in the supplemental information (Tables S1 and S2). For new interactions introduced in the integrated model—such as VEGF-induced Tie2 shedding, S1P-induced Ang2 release, and Tie2-mediated downstream activation of Akt and sequestration of Src—parameter values were estimated by fitting simulation outputs to time-course experimental data. We first used manual tuning to obtain initial estimates for the new parameters were hand-tuned to roughly match the trends and timescales observed in the experimental datasets. To obtain the final set of parameters, we use a metaheuristic optimization Pattern Search algorithm, implemented via the Global Optimization Toolbox in MATLAB R2022a that minimizes the sum of squared errors between the model simulations and experimental measurements across multiple observables.^88^ Experimental data measuring the constitutive shedding of Tie2, VEGF-induced extracellular domain shedding of Tie2,^31^ VEGF-induced activation of S1P,^37^ and S1P-induced Ang2 release dynamics^36^ are used for model calibration. Experimental data on the effect of chronic Ang1 stimulation on VEGF-induced Src and VE-Cadherin activation^35^ are used for model validation.

#### Global parameter sensitivity analysis

To identify parameters and underlying molecular mechanisms that affect the activation of Akt and Src, we perform global sensitivity analysis using Sobol indices, focusing on both first-order (main-effect) and second-order (synergy) indices. The parameter space is sampled using Latin hypercube sampling (LHS), and the Sobol indices for all model parameters are calculated as described in the STAR Methods section. The model parameters are varied from 0.2-fold to 5-fold of their baseline values and randomly sampled using LHS to evaluate their effects on the signaling outcomes pVEGFR2, pTie2, ppAkt and pSrc at 5 minutes post-stimulation with 10 ng/mL VEGF, 200 ng/mL Ang1, and 500 ng/mL Ang2. A complete list of model parameters, along with their descriptions, is available in the supplemental information (Table S1).

The first-order Sobol analysis involved 30,000 total runs to quantify the individual contributions of parameters to the variance of the signaling outcomes pVEGFR2, pTie2, ppAkt, and pSrc at 5 minutes post-stimulation with 10 ng/mL VEGF, 200 ng/mL Ang1, and 500 ng/mL Ang2. Bootstrapping was employed to calculate 95% confidence intervals for the first-order Sobol indices, ensuring statistical rigor in the analysis. Additionally, to assess parameter interactions, we performed second-order Sobol analysis with 112,000 runs for each of the four observables, providing comprehensive insights into synergistic effects between parameter pairs. The Sobol sequence was configured with a skip value of 10ˆ3 and a leap value of 10ˆ2, balancing computational efficiency and coverage of the parameter space.

The analysis is performed using MATLAB R2022a (MathWorks, Natick, MA). The analysis is parallelized and performed using the high-performance computing resources at the Advanced Research Computing at Hopkins (ARCH) core facility (rockfish.jhu.edu).

### Quantification and statistical analysis

Bootstrapping was employed to estimate 95% confidence intervals for Sobol indices, and the same random seed was used across all simulations to ensure reproducibility.

In our first-order Sobol analysis: 30,000 simulations were performed, and 112,000 simulations were performed for second-order Sobol analysis using MATLAB R2022a for data processing and visualization.

## Supplemental information

Document S1. Table S2

Table S1. List of all model parameters, descriptions, and their best-fit values

Data S1. Computational model of the endothelial signaling network formed by VEGF and Ang-Tie pathways, in SBML, related to Figure 1
