# Supplementary material for: Promoting vascular stability through Src inhibition and Tie2 activation: A model-based analysis
Source: iScience. 2025 May 9;28(6):112625. doi: 10.1016/j.isci.2025.112625 (PMC12148613; doi:10.1016/j.isci.2025.112625)
Supplement: Document S1. Table S2 [file mmc1.pdf]

iScience, Volume *28*

Supplemental information

Promoting vascular stability through Src inhibition and Tie2 activation: A model-based analysis

Yu Zhang, Christopher D. Kontos, Brian H. Annex, and Aleksander S. Popel

Supplemental Information

Table S2. List of all model initial conditions

Parameter	Description	Value	Unit
'Tie2_0'	Initial concentration of Tie2	1.00E-02	nM
'Ang1_4_0'	Initial concentration of tetrameric Ang1	Varies	nM
'Ang2_2_0'	Initial concentration of dimeric Ang2	Varies	nM
'Ang2_3_0'	Initial concentration of trimeric Ang2	Varies	nM
'Ang2_4_0'	Initial concentration of tetrameric Ang2	Varies	nM
'VEPTP_0'	Initial concentration of VE-PTP	1.00E+03	nM
'sTie2_0'	Initial concentration of soluble Tie2	0	nM
'sTie1_0'	Initial concentration of sobuble Tie1	0	nM
'Tie1_0'	Initial concentration of membrane Tie1	5.00E-03	nM
'Tie1Tie2_0'	Initial concentration of Tie1-Tie2 heterodimer	5.00E-03	nM
'VEGF_0'	Initial concentration of VEGF	0	μM
'VEGFR1_0'	Initial concentration of VEGFR1	1.43	#/μm2
'VEGFR2_0'	Initial concentration of VEGFR2	4.29E+00	#/μm2
'NRP1_0'	Initial concentration of neuropilin 1	2.86E+01	#/μm2
'CD47_0'	Initial concentration of CD47	7.14E+00	μM
'TSP1_0'	Initial concentration of TSP1	0.00E+00	μM
'PLCgamma_0'	Initial concentration of PLC gamma	0.2	μM
'PKC_0'	Initial concentration of PKC	0.1	μM
'CIB1_0'	Initial concentration of CIB1	0.5	μM
'SphK_0'	Initial concentration of sphingosine kinase	1.00E-01	μM
'Sph_0'	Initial concentration of sphingosine	10	μM
'S1P_0'	Initial concentration of sphingosine-1-phosphate	0	μM
'RasGTP_0'	Initial concentration of RasGTP	0	μM
'Raf_0'	initial concentration of Raf	0.355472	μM
'MEK12_0'	Initial concentration of MEK1/2	0.288919	μM
'ERK12_0'	Initial concentration of ERK1/2	0.38233	μM
'Tie1Protease_0'	Initial concentration of Tie1 protease	0.1	μM
'Calcium_0'	Initial concentration of calcium	0.05	μM
'CaER_0'	Initial concentration of CaER	2.00E+03	μM
'CaF_0'	Initial concentration of CaF	118	μM
'CaM_0'	Initial concentration of CaM	1	μM
'Istim0'	Initial concentration of Istim	0.18	μM
'Axl_0'	Initial concentration of Axl	7.14	#/μm2
'PTEN_0'	Initial concentration of PTEN	0.1	μM
'PI_0'	Initial concentration of PI	10	μM
'PI3K_0'	Initial concentrationof PI3K	0.1	μM
'Akt_0'	Initial concentraion of Akt	0.1	μM
'PDK1_0'	Initial concentration of PDK	0.1	μM
'Tie2Protease_0'	Initial concentrationof Tie2 protease	0.1	μM

RhoA_0'	Initial concentraion of RhoA	0.1	μM
'mDia_0'	Initial concentrationof mDia	0.1	μM
'Src_0'	Initial concentration of Src	0.1	μM
'VECadherin_0'	Initial concentration of VE-Cadherin	0.1	μM
'eNOS_0'	Initial concentration of eNOS	0.1	μM
p38_0'	Initial concentration of p38	0.1	μM
